# Personalized neurometabolic signature predicts seizure outcomes of laser ablation in mesial temporal lobe epilepsy

**DOI:** 10.1038/s43856-025-01167-0

**Published:** 2025-11-20

**Authors:** Jiajie Mo, Baotian Zhao, Xiu Wang, Chao Zhang, Lin Sang, Wenhan Hu, Xiaoqiu Shao, Jianguo Zhang, Rong Li, Kai Zhang

**Affiliations:** 1 Department of Neurosurgery, Beijing Tiantan Hospital, Capital Medical University, Beijing, China; 2 Department of Neurosurgery, Beijing Fengtai Hospital, Beijing, China; 3 Department of Neurosurgery, Beijing Neurosurgical Institute, Capital Medical University, Beijing, China; 4 Department of Neurology, Beijing Tiantan Hospital, Capital Medical University, Beijing, China; 5 MRI Research Center, Key laboratory for NeuroInformation of Ministry of Education, School of Life Science and Technology, University of Electronic Science and Technology of China, Chengdu, China

**Keywords:** Prognostic markers, Prognostic markers, Epilepsy

## Abstract

**Background:**

Mesial temporal lobe epilepsy (mTLE) is a common form of drug-resistant epilepsy and seizure outcomes after minimally invasive laser ablation remain suboptimal. Current imaging-guided strategies often fail to capture individual variability in seizure foci. This study aimed to develop a personalized neuroimaging biomarker to improve surgical planning and predict outcomes.

**Methods:**

Thirty patients with mTLE (16 women, 53.3%; age range 17–59 years) who underwent magnetic resonance-guided laser interstitial thermal therapy were retrospectively analyzed. The asymmetry index (AI) from [^18^F]fluorodeoxyglucose positron emission tomography ([^18^F]FDG PET) defined the personalized NeuroMetabolic Signature (pNMS). Prognostic thresholds and optimal pNMS ablative rate were explored using restricted cubic spline (RCS) analysis and Youden’s index as statistical methods for identifying cutoffs. A generalized additive model (GAM) was applied to examine imaging-derived features associated with pNMS.

**Results:**

Here we show that the AI of PET metabolic values significantly predicted seizure outcomes (odds ratio = 1.43, *P* = 0.02), with −0.06 as the threshold for defining pNMS (*P* for non-linearity = 0.04). A hippocampal pNMS ablative rate of 39.79% is significantly associated with seizure freedom (Pearson *χ*^*2*^ = 10.16, *P* = 0.001; balanced accuracy = 0.83). Hippocampal atrophy contributes most to pNMS expression (Shapley value = −0.026), and correlates with metabolic asymmetry (Pearson’s *r* = 0.47, *P* < 0.01).

**Conclusions:**

The pNMS provides an individualized imaging marker for guiding laser ablation and predicting postoperative seizure outcomes. This approach supports more precise surgical planning and may improve long-term prognosis in patients with mesial temporal lobe epilepsy.

## Abstract (web summary)

Mo et al. develop a Personalized NeuroMetabolic Signature (pNMS) from [18F]FDG PET imaging to guide laser ablation in mesial temporal lobe epilepsy. The pNMS offers clinicians a practical tool for surgical planning and helps improve seizure outcomes for patients.

## Plain language summary

Mesial temporal lobe epilepsy (mTLE) is a common type of refractory epilepsy that does not improve with medicines. One treatment option is a minimally invasive procedure called laser ablation that destroys the brain tissue causing seizures. However, predicting who will benefit from this surgery remains difficult. In this study, we developed a Personalized NeuroMetabolic Signature (pNMS) using [^18^F]fluorodeoxyglucose positron emission tomography ([^18^F]FDG PET) imaging, which shows areas of abnormal metabolism. We studied 30 patients and found that the pNMS could predict seizure outcomes after surgery and identify how much abnormal tissue needed to be removed. These findings suggest that pNMS may facilitate improved laser trajectory planning for clinicians and enhance patients’ surgical outcomes in the future.

## Introduction

Mesial temporal lobe epilepsy (mTLE) is one of the most prevalent forms of drug-resistant epilepsy, necessitating the development of advanced therapeutic interventions^1^. Magnetic resonance–guided laser interstitial thermal therapy (MRgLITT) has emerged as a minimally invasive surgical technique that is both effective and safe for treating epilepsy^2,3^. Studies have reported seizure freedom rates of approximately 60% following MRgLITT, with complete ablation of the epileptogenic zone (EZ) identified as a critical determinant of successful outcomes^4^. Historically, structural imaging-guided ablation strategies have primarily targeted mesial temporal structures, such as the amygdala, hippocampus and piriform cortex^5^. However, these approaches may not adequately capture individual variability in seizure foci^6^. The application of neuroimaging biomarkers to guide optimized and personalized ablation strategies remains largely unexplored. Therefore, there is a critical need to develop individualized imaging-based approaches to improve surgical outcomes in patients with mTLE.

In the presurgical evaluation of epilepsy, [^18^F]fluorodeoxyglucose positron emission tomography ([^18^F]FDG PET) imaging has become indispensable for localizing and delineating epileptogenic foci owing to its ability to detect abnormal metabolic changes associated with seizure activity^7,8^. Recent studies have demonstrated that quantitative analysis offers greater precision and objectivity than traditional qualitative visual interpretation^9^. Moreover, advances in identifying interhemispheric metabolic asymmetries on PET images have introduced a more optimized and personalized approach for delineating the EZ^10^. This strategy may facilitate improved laser trajectory planning and enhance the surgical outcomes.

In this study, we develop a Personalized NeuroMetabolic Signature (pNMS), quantified as the asymmetry index (AI) of PET metabolic values, predicts seizure outcomes after MRgLITT in mTLE. We demonstrate that pNMS identifies patient-specific abnormal regions, that the extent of pNMS ablation is associated with seizure freedom, and that hippocampal atrophy is a key structural correlate of pNMS. These findings indicate that pNMS provides a clinically relevant imaging marker to guide individualized ablation strategies and improve surgical outcomes in mTLE.

## Methods

### Participants

This retrospective cohort study consecutively included patients diagnosed with mTLE who underwent MRgLITT between August 2020 and December 2023 at Beijing Tiantan Hospital. Each patient diagnosed as mTLE after a comprehensive presurgical evaluation, including long-term video-scalp electroencephalography (EEG) monitoring, multimodalities brain MRI [T1-weighted magnetization-prepared rapid acquisition gradient echo (T1WI MPRAGE); T2-weighted turbo spin echo (T2WI TSE); T2-weighted fluid-attenuated inversion recovery (T2WI FLAIR)], and [^18^F]FDG PET/CT. Noninvasive data were reviewed at multidisciplinary epilepsy conferences to reach a consensus on the most likely location of the EZ and to determine whether the patient should proceed directly to surgery or undergo intracranial stereoelectroencephalography (SEEG). SEEG implantation was based on the concept of an epileptic “network”, a set of anatomically and functionally interconnected structures. This approach allows for anatomo-clinical-electrical correlations, in which the electroclinical features of seizures are analyzed in relation to the specific anatomical regions sampled.

Eligible patients met the following criteria: (i) refractory epilepsy, defined as failure of adequate trials of at least two tolerated, appropriately chosen, and properly used antiepileptic drug schedules for a minimum of six months^11^; (ii) localization of the EZ in the temporal lobe based on detailed presurgical assessments; (iii) agreement to undergo MRgLITT and provision of informed consent by the patient or legal guardian; and (iv) availability of more than 12 months of postoperative follow-up. The exclusion criteria were as follows: (i) poor-quality neuroimaging data, such as motion artifacts, aliasing, or rippling related to eye movement; (ii) history of reoperation; and (iii) incomplete clinical information.

The study followed the Strengthening the Reporting of Observational Studies in Epidemiology (STROBE) statement^12^ and adhered to the principles of the Declaration of Helsinki and its subsequent amendments. The Institutional Review Board of Beijing Tiantan Hospital (KY2020-126-01) approved anonymized data collection and data sharing prior to the study’s commencement. All patients or their guardians provided written general informed consent to participate in the study.

### Data sources and measurement

Demographic data were collected from the patients’ medical records. Postoperative seizure outcomes at 1 year were assessed using the International League Against Epilepsy (ILAE) classification system^13^. Patients classified as ILAE class 1 (completely seizure-free without auras) were considered seizure-free (SF), while those classified in any other ILAE class were categorized as not seizure-free (NSF). Prior to MRgLITT, all patients underwent magnetic resonance imaging according to a standard epilepsy protocol. All structural MRI scans were acquired on a 3 T Siemens Verio scanner. The T1WI MPRAGE sequence parameters were: repetition time (TR) = 2300 ms, echo time (TE) = 2.53 ms, flip angle = 12°, slice thickness = 1 mm, no gap, voxel size = 1.0 × 1.0 × 1.0 mm^3^. The T2WI FLAIR sequence was acquired with: TR = 7000 ms, TE = 80 ms, flip angle = 12°, slice thickness = 1 mm, no gap, voxel size = 1.5 × 1.5 × 1.5 mm^3^. Interictal [^18^F]FDG PET scans were performed under standardized resting conditions using the GE Discovery ST PET-CT system (field of view = 300 mm, matrix = 192 × 192, slice thickness = 3.27 mm). Patients were instructed to rest quietly in a dimly lit room during the 40 min following the intravenous administration of ^18^F-FDG at a mean dose of 310MBq/70 kg body weight. PET images were reconstructed using the ordered subset expectation maximization (OSEM) algorithm with 16 subsets and 6 iterations. Additionally, all patients underwent [^18^F]FDG PET imaging under routine resting conditions within six months of presurgical evaluation, with no clinical seizures reported within six hours before or during the scan^14^.

### Imaging preprocessing procedures

**Fig. 1:**
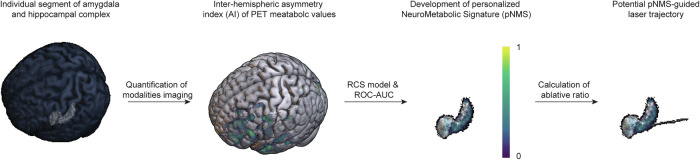
Summary of the methodological approach. Thirty patients with mesial temporal lobe epilepsy (mTLE) who underwent magnetic resonance-guided laser interstitial thermal therapy (MRgLITT) were evaluated. This study leveraged inter-hemispheric asymmetry index (AI) of PET metabolic values to develop personalized NeuroMetabolic Signature (pNMS) for guiding optimal ablative range and support pNMS-guided laser trajectory planning in clinical practice. Abbreviations: AI asymmetry index, RCS restricted cubic spline, ROC-AUC receiver operating characteristic-area under the curve.

To enhance treatment precision and improve surgical outcomes, the study pursued three key objectives: (i) to propose a framework for deriving modality-specific quantitative thresholds for developing the pNMS; (ii) to explore an approach for estimating the optimal ablative rate of pNMS associated with seizure freedom; (iii) to interpret the biological underpinnings of pNMS through analysis of structural variables (Fig. 1).

Image data preprocessing included the following steps: (ⅰ) a classical unified segmentation approach was applied to segment the T1WI images into gray matter, white matter, and cerebrospinal fluid, generating an explicit mask for subsequent analysis; (ⅱ) T2WI FLAIR, [^18^F]FDG PET images, and postoperative T1WI images were coregistrated to the preoperative T1WI MPRAGE images for each subject; (ⅲ) ablative mask was manually delineated slice by slice on the postoperative contrast-enhanced T1WI MPRAGE images using MRIcron software (https://www.mricro.com). Two experts independently segmented surgical cavities on co-registered post-contrast T1WI images, achieving intra- and inter-rater Sørensen–Dice similarity coefficient [Dice(A,B) = 2*|intersection(A,B)|/( | A | + | B | ) (A: 1st label, B: 2nd label)] of 0.85 ± 0.03 and 0.83 ± 0.04, respectively; (ⅳ) All images were normalized to the Montreal Neurological Institute (MNI) standard space using the ‘Normalize’ module implemented in SPM12 with visually inspection for quality; (ⅴ) To improve the signal-to-noise ratio, the PET images were smoothed using a Gaussian kernel with a full width at half maximum of 6 mm. Additionally, to address quantitative inaccuracies arising from the inherently limited spatial resolution of PET imaging, partial volume effects correction was performed using the voxel-wise three-compartment Müller–Gärtner method^15^; (ⅵ) gray matter densities, FLAIR intensity signals, and PET metabolic values of each modality were normalized using proportional scaling to mitigate interindividual variability. Specifically, within the brain mask, voxel-wise values were standardized by subtracting the mean and dividing by the standard deviation of each participant’s voxel distribution, facilitating robust quantitative comparisons across subjects and modalities (Supplementary Fig. 1A); (ⅶ) Processed images were flipped along the midsagittal plane using the ‘ImCalc’ module in SPM12 with the expression ‘flipud(i1)’. The AI for each modality was calculated using the formula “(i1-i2)./((i1 + i2).*0.5)” (Supplementary Fig. 1B). Quantitative variables for pNMS, calculated using AI of multimodal images, were processed using SPM12 (https://www.fil.ion.ucl.ac.uk/spm) running on MATLAB 2021a software (The MathWorks Inc., Natick, MA, USA).

### Investigation of the relationship between quantitative variables and seizure outcomes

The construction of the pNMS involved two key steps: (1) the selection of imaging modalities with prognostic value based on group-level differences and (2) the determination of prognostic thresholds for each modality. These steps were carried systematically in a data-driven manner to ensure that the pNMS accurately reflected the imaging features associated with seizure outcomes.

First, the AI of quantitative imaging variables (including gray matter densities, FLAIR intensity signals, and PET metabolic values) within the ablation mask were compared between the SF and NSF groups. Modalities showing significant group-level differences were identified as having prognostic potential and were selected for inclusion in the pNMS. Additionally, logistic regression analysis was employed to assess the association between clinical features (side of seizure focus, stereoelectroencephalography (SEEG) implantation, age at seizure onset, and epilepsy duration) and imaging features (T1WI gray matter densities, FLAIR intensity signals, and PET metabolic values) with seizure outcomes. The results are presented as odds ratios (ORs) with 95% confidence intervals (CIs) and corresponding *P* values. An OR greater than 1 indicated a higher likelihood of achieving seizure freedom.

Second, to precisely define prognostic thresholds for each selected imaging modality, the nonlinear association between quantitative variables (gray matter densities, FLAIR intensity signals, and PET metabolic values) and seizure outcomes was analyzed using restricted cubic spline (RCS) analysis within a logistic regression framework. Adjustments were made for clinical variables, including the side of the EZ, SEEG implantation, and age at seizure onset. The RCS model with the lowest Bayesian information criterion (BIC) value was selected for final analysis. The RCS included three knots at the 10th, 50th and 90th percentiles. The reference values were determined based on the shape of the RCS curve. In cases where the curve exhibited U-, N-, or L-shaped patterns, the inflection points (i.e., points where the curve changed direction) were set as the reference thresholds. The resulting pNMS integrates the selected modalities and their corresponding prognostic thresholds, grounded in imaging features significantly associated with seizure outcomes (Supplementary Fig. 1C).

### Determination of the optimal ablative rate of pNMS for achieving seizure freedom

First, the overlay rates between the pNMS and ablation mask in the ipsilateral amygdala and hippocampus were compared between the SF and NSF groups. Segmentation of the amygdala and hippocampus was performed using the intrinsic *segmentHA_T1* module of *FreeSurfer* software^16^ (https://surfer.nmr.mgh.harvard.edu/pub/dist/freesurfer/dev/). The ablative rate was defined as the ratio of the overlapping volume (intersection of pNMS and ablation mask) to the total volume of the pNMS, representing the proportion of patient-specific metabolic abnormalities that were successfully ablated. Second, the optimal pNMS ablative rate associated with seizure freedom was evaluated using the RCS model. To validate the findings, Youden’s index was calculated to identify the optimal threshold, and the predictive accuracy of the pNMS ablative rate was assessed using receiver operating characteristic-area under the curve (ROC-AUC), sensitivity, and specificity metrics. Considering the class imbalance in our dataset, where the rate of seizure-free patients was approximately 70%, we used the Balanced Accuracy (BAcc) metric, defined as the arithmetic mean between sensitivity and specificity. This provides a more reliable performance evaluation for imbalanced data, as it accounts for the performance on both classes in the presence of imbalance^17^.

### Exploration of predictors for Neurometabolic abnormalities

The total intracranial volume (TIV), ipsilateral volumetric measurements and volumetric AI of the amygdala and hippocampus were calculated for all patients to predict and interpret the alteration in the pNMS using a generalized additive model (GAM). Five-fold cross-validation strategy was implemented across 1,000 iterations to ensure a robust performance evaluation, with 95% CIs computed around each prediction. To assess feature importance within the model, Shapley values and partial dependence plots were evaluated to identify their overall contributions. In addition, Pearson’s correlation analysis was used to quantify the relationship between the actual and predicted values of pNMS expression, serving as a measure of predictive accuracy (Supplementary Fig. 1D).

### Statistics and reproducibility

Continuous variables are presented as mean ± standard deviation (SD) or median with interquartile range (IQR), as appropriate. Categorical variables were expressed as counts (percentages). Differences between continuous variables in the two independent groups were assessed using the Student’s *t* test or Wilcoxon rank-sum test, as appropriate. Categorical variables were compared using Pearson’s *χ*^*2*^ test, the Likelihood-ratio *G*^*2*^ test, or Fisher’s exact test, depending on the expected cell sizes. Analysis of covariance (ANCOVA) was used to compare the differences between group means adjusted for the covariate. Pearson’s correlation coefficients were calculated to assess the linear associations between continuous variables. All statistical tests were two-sided, with a significance level set at *α* = 0.05. *P* < 0.05 was considered statistically significant. All statistical analyses were conducted using *R* software version 4.3.0 (https://www.r-project.org/).

### Ethics approval

The Institutional Review Board of Beijing Tiantan Hospital (KY2020-126-01) approval for anonymized data collection and data sharing prior to the study’s commencement. All patients or their guardians gave written general informed consent for participating in scientific studies.

## Results

### Demographic

A total of 30 patients with mTLE who underwent MRgLITT were included in the final analysis, all of whom met the inclusion criteria of this study. Among them, 21 patients (70.0%) achieved seizure freedom at the 1-year postoperative follow-up visit. The cohort consisted of 16 females (53.3%), with a mean age at seizure onset of 21.84 ± 8.63 years, median epilepsy duration of 10 years (IQR 5.00–18.00), and mean age at surgery of 33.87 ± 11.20 years. Regarding lateralization, 18 patients (60.0%) had a left-sided EZ. Twenty-nine (96.7%) patients experienced loss of consciousness during seizures. Scalp EEG revealed interictal and ictal focal/regional discharge patterns in 15 (46.7%) and 21 (70.0%) patients, respectively. Additionally, 29 patients (96.7%) showed increased FLAIR signals and PET hypometabolism. There were no significant differences in demographic variables between the SF and NSF groups (Table 1).

**Table 1:** Demographic

Variables	Total (*n* = 30)	NSF (*n* = 9)	SF (*n* = 21)	Statistic
Gender, *n*(%)				Fisher’s Exact, *P* = 0.69
Female	16 (53.33)	4 (44.44)	12 (57.14)	
Male	14 (46.67)	5 (55.56)	9 (42.86)	
Side of EZ, *n* (%)				Fisher’s Exact, *P* = 0.70
Right	12 (40.00)	3 (33.33)	9 (42.86)	
Left	18 (60.00)	6 (66.67)	12 (57.14)	
Age at seizure onset, mean ± SD	21.84 ± 8.63	23.76 ± 11.80	21.01 ± 7.07	Student’s *t* = 0.79, *P* = 0.43
Epilepsy duration, median (Q₁, Q₃)	10.00 (5.00, 18.00)	11.00 (7.00, 20.00)	7.00 (5.00, 18.00)	Mann–Whitney *U* = −0.59, *P* = 0.56
Age at surgery, mean ± SD	33.87 ± 11.20	37.67 ± 12.67	32.24 ± 10.41	Student’s *t* = 1.23, *P* = 0.23
IID, *n* (%)				Fisher’s Exact, *P* = 1.00
Bilateral/Diffuse	16 (53.33)	5 (55.56)	11 (52.38)	
Focal/Regional	14 (46.67)	4 (44.44)	10 (47.62)	
ID, *n* (%)				Fisher’s Exact, *P* = 0.68
Bilateral/Diffuse	9 (30.00)	2 (22.22)	7 (33.33)	
Focal/Regional	21 (70.00)	7 (77.78)	14 (66.67)	
Aura, *n* (%)				Fisher’s Exact, *P* = 1.00
No	7 (23.33)	2 (22.22)	5 (23.81)	
Yes	23 (76.67)	7 (77.78)	16 (76.19)	
LOC, *n* (%)				Fisher’s Exact, *P* = 0.30
No	1 (3.33)	1 (11.11)	0 (0.00)	
Yes	29 (96.67)	8 (88.89)	21 (100.00)	
Automatism, *n* (%)				Fisher’s Exact, *P* = 0.56
No	4 (13.33)	2 (22.22)	2 (9.52)	
Yes	26 (86.67)	7 (77.78)	19 (90.48)	
ICDP, *n* (%)				Fisher’s Exact, *P* = 0.68
No	21 (70.00)	7 (77.78)	14 (66.67)	
Yes	9 (30.00)	2 (22.22)	7 (33.33)	
F2BTCS, *n* (%)				Fisher’s Exact, *P* = 0.43
No	15 (50.00)	3 (33.33)	12 (57.14)	
Yes	15 (50.00)	6 (66.67)	9 (42.86)	
HA, *n* (%)				Fisher’s Exact, *P* = 0.67
No	8 (26.67)	3 (33.33)	5 (23.81)	
Yes	22 (73.33)	6 (66.67)	16 (76.19)	
IHI, *n* (%)				Fisher’s Exact, *P* = 0.67
No	20 (66.67)	7 (77.78)	13 (61.90)	
Yes	10 (33.33)	2 (22.22)	8 (38.10)	
TP atrophy/blurring, *n* (%)				Fisher’s Exact, *P* = 1.00
No	12 (40.00)	4 (44.44)	8 (38.10)	
Yes	18 (60.00)	5 (55.56)	13 (61.90)	
Increased FLAIR signal, *n* (%)				Fisher’s Exact, *P* = 1.00
No	1 (3.33)	0 (0.00)	1 (4.76)	
Yes	29 (96.67)	9 (100.00)	20 (95.24)	
^18^FDG-PET hypometabolism, *n* (%)				Fisher’s Exact, *P* = 1.00
No	1 (3.33)	0 (0.00)	1 (4.76)	
Yes	29 (96.67)	9 (100.00)	20 (95.24)	
SEEG implantation, *n* (%)				Fisher’s Exact, *P* = 0.20
No	22 (73.33)	5 (55.56)	17 (80.95)	
Yes	8 (26.67)	4 (44.44)	4 (19.05)	

Continuous variables are presented as mean ± standard deviation or median (1st quartile, 3rd quartile), as appropriate. Between-group comparisons were performed using the two-sided Student’s *t* test or Wilcoxon rank-sum test for continuous variables, and Pearson’s *χ²* test or Fisher’s exact test for categorical variables, as appropriate.

*n* number of individuals in each cohort, *SF* seizure free, *NSF* non-seizure free, *EZ* epileptogenic zone, *IID* interictal discharges, *ID* ictal discharges, *LOC* loss of consciousness, *ICDP* ictal contralateral dystonic posturing, *F2BTCS* focal to bilateral tonic-clonic seizures, *HA* hippocampal atrophy, *IHI* incomplete hippocampal inversion, *TP* temporal pole, *FLAIR* fluid-attenuated inversion recovery, ^18^*FDG-PET* ^18^fluorodeoxyglucose-positron emission tomography, *SEEG* stereoelectroencephalography.

### Leveraging PET metabolic values to develop pNMS for guiding optimal ablation

**Fig. 2:**
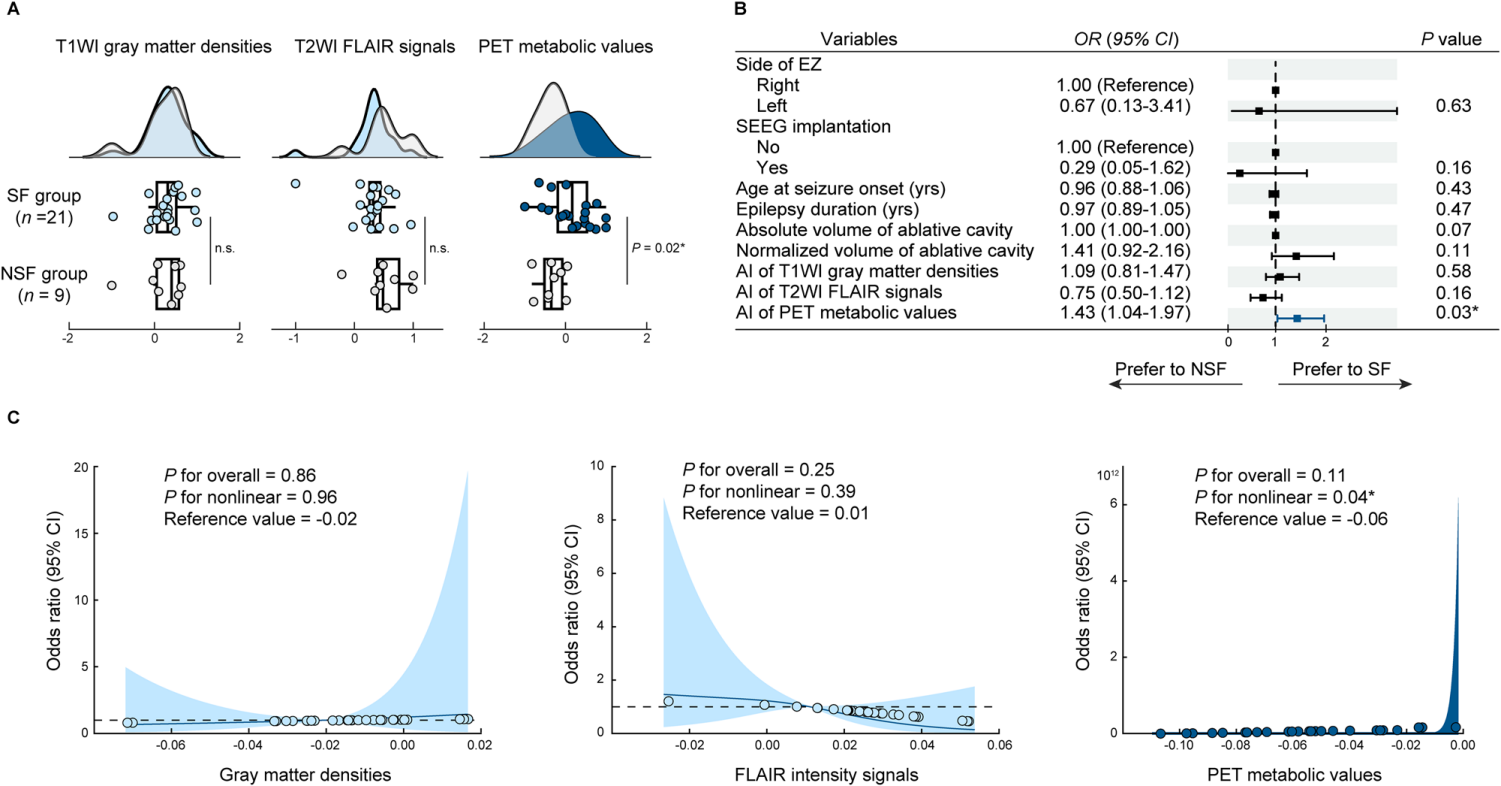
Relationship between quantitative neuroimaging variables and seizure outcomes. **A** Comparison of the asymmetry index (AI) of multimodalities within the ablative range mask between seizure freedom (SF, *n* = 21) and not SF (NSF, *n* = 9) groups. *P* values were calculated using two-sided Student’s *t* test. In the boxplots, the center line represents the mean value, the hinges represent the standard deviation (SD); **B** Forest plot showing results of univariate logistic regression analyses of clinical and imaging predictors for SF in the full cohort (*n* = 30). The vertical lines inside the boxes represent the odds ratio (OR) estimates and the horizontal error bars represent the 95% confidence intervals (CIs). Models were two-sided and unadjusted for multiple comparisons; **C** Restricted cubic spline (RCS) analysis based on logistic regression was used to determine the threshold of the AI of each imaging modality associated with SF. The solid line represents the estimated OR, and the shaded bands indicate the 95% CIs. Analyses were two-sided and unadjusted for multiple comparisons. * Indicates statistical significance at *P* < 0.05. Abbreviations: FLAIR fluid-attenuated inversion recovery, PET positron emission tomography, SF seizure-free, NSF not seizure-free, EZ epileptogenic zone, SEEG stereoelectroencephalography, AI asymmetry index.

We first compared the AI of multimodality neuroimaging values within the ablation mask between the SF and NSF groups. No statistically significant differences in T1WI gray matter densities (Mann–Whitney *U* = −0.00, *P* = 1.00) or T2WI FLAIR signals (Mann–Whitney *U* = −1.96, *P* = 0.05), whereas a statistically significant difference was observed in PET metabolic values (Student’s *t* = −2.59, *P* = 0.02; Fig. 2A). These findings remained robust after adjusting for TIV (*F* = 4.38, *P* = 0.02), volumes of hippocampus and amygdala (*F* = 8.85, *P* = 0.001), and AI of hippocampus and amygdala (*F* = 4.25, *P* = 0.03; Supplementary Table 1).

We then explored the predictors of seizure freedom using univariate logistic regression analysis. As shown in Supplementary Table 2, variables including the side of the EZ, SEEG implantation, age at seizure onset, epilepsy duration, absolute volume of the ablative cavity, normalized volume of the ablative cavity (adjusted for TIV), the AI of T1WI gray matter densities, and T2WI FLAIR signals were not statistically significant predictors of seizure freedom. In contrast, PET metabolic values were statistically associated with seizure freedom, with higher AI values predicting a greater likelihood of favorable outcomes (OR with 95% CI = 1.43, 1.04–1.97, *P* = 0.03; Fig. 2B). These results support the AI of PET metabolic values as a potential imaging biomarker for predicting seizure outcomes.

RCS analysis demonstrated that AI values derived from T1WI gray matter densities and T2WI FLAIR signals exhibited non-significant U-shaped associations with seizure freedom (*P* for non-linearity = 0.96 and 0.39, respectively), with inflection points observed at −0.02 and 0.01. Conversely, PET metabolic values showed a statistically significant L-shaped association with seizure freedom (*P* for non-linearity = 0.04), with an inflection point at −0.06 (Fig. 2C). Patients with higher PET metabolic values beyond this threshold were significantly more likely to achieve seizure freedom after MRgLITT.

Based on this, we defined −0.06 as the threshold for the AI of PET metabolic values to construct binarized pNMS. This binarized pNMS were then used to guide the identification of the optimal ablative range for achieving seizure freedom.

### Determining the optimal ablative rate of pNMS for achieving seizure freedom

**Fig. 3:**
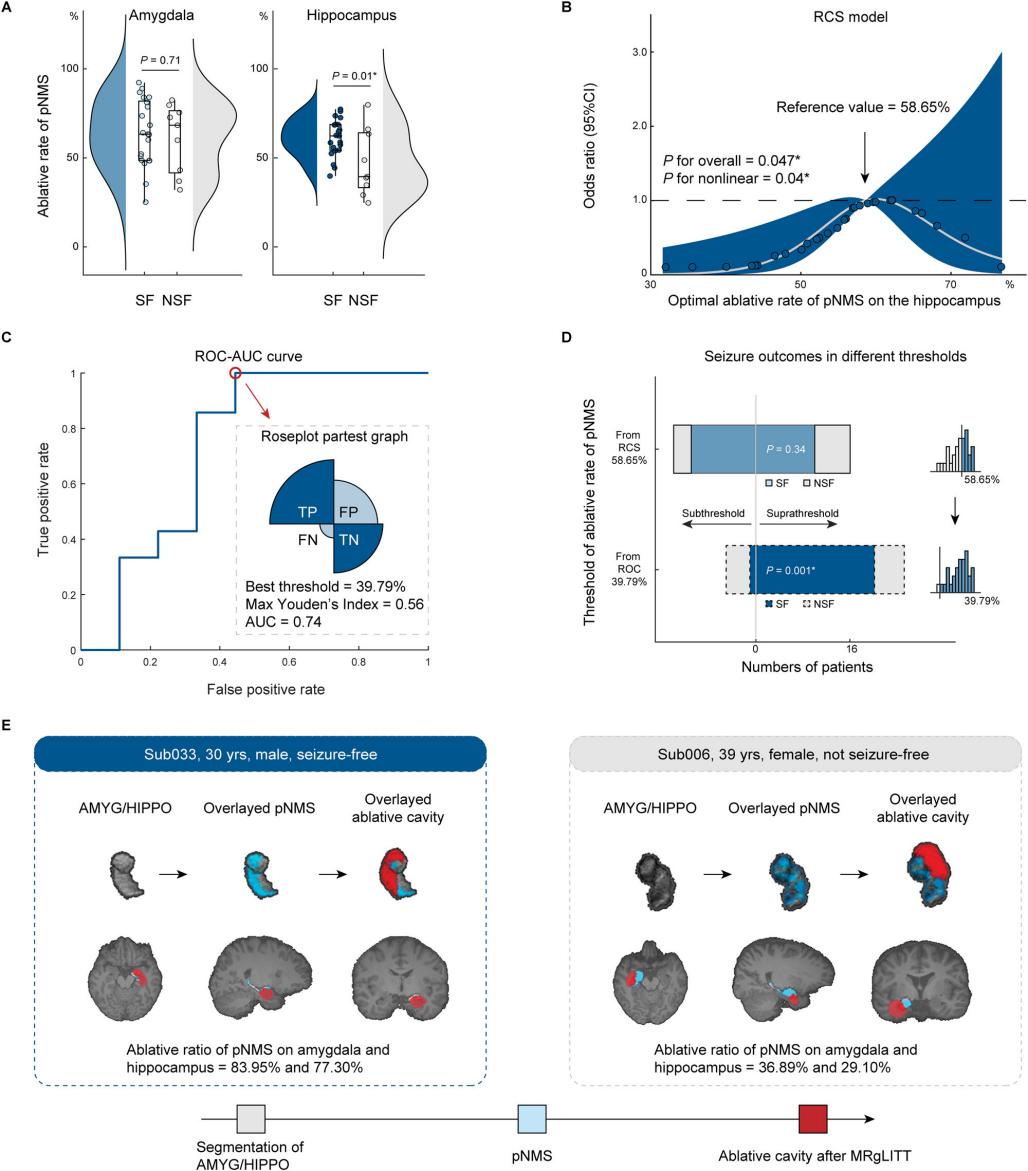
Determination of the optimal ablative rate of personalized NeuroMetabolic Signature (pNMS). **A** Comparison of the overlay rates between pNMS and ablative range within the ipsilateral amygdala and hippocampus between seizure-freedom (SF, *n* = 21) and not seizure-freedom (NSF, *n* = 9) groups. *P* values were calculated using two-sided Student’s *t* test (amygdala: *P* = 0.71; hippocampus: *P* = 0.01). In the boxplots, the center line represents the mean value, the hinges represent the standard deviation (SD); **B** Restricted cubic spline (RCS) analysis illustrating the nonlinear relationship between the ablative rate of pNMS on the hippocampus and seizure freedom. A significant L-shaped association was observed, with an inflection point (reference value) at 58.65% (*P* for overall = 0.047; *P* for non-linearity = 0.04). The solid line represents the odds ratio, and the shaded area indicates the 95% confidence interval (CI) of the estimate (two-sided logistic regression, no multiple-comparison correction); **C** Receiver operating characteristic-area under the curve (ROC-AUC) analysis used to determine the optimal threshold of the hippocampal pNMS ablative rate for seizure outcome prediction, evaluated by the maximum Youden’s index; **D** Seizure freedom rates in patients stratified by the RCS-derived threshold (58.65%, *P* = 0.34) and Youden-derived threshold (39.79%, *P* = 0.001) of hippocampal pNMS ablative rate. *P* values were calculated using two-sided Pearson’s *χ²* test without multiple-comparison correction; **E** Overlay relationship between the pNMS distribution and the ablative cavity in two representative cases with different seizure outcomes. All analyses in (**B**–**D**) were performed in the full cohort (*n* = 30). Gray, blue and red ranges indicated the overlay relationship between individual segmentation of amygdala and hippocampus, pNMS, and ablative cavity after magnetic resonance-guided laser interstitial thermal therapy (MRgLITT). * Indicates statistical significance at *P* < 0.05. Abbreviations*:* TP true positive, FP false positive, FN false negative, TN true negative, AMYG amygdala, HIPPO hippocampus.

The overlay rates between pNMS and the ablation mask within the ipsilateral hippocampus was significantly higher in the SF group than in the NSF group (Student’s *t* = −2.61, *P* = 0.01), and no statistical significance was found in the ipsilateral amygdala (Student’s *t* = −0.38, *P* = 0.71; Fig. 3A). RCS analysis revealed a significant U-shaped association between the ablative rate of pNMS in the hippocampus and seizure freedom (*P* for overall = 0.047; *P* for non-linearity = 0.04), with an inflection identified at 58.65% (Fig. 3B). This suggests that a higher pNMS ablative rate beyond this value is associated with an increased likelihood of seizure freedom following MRgLITT. To further assess predictive utility, ROC analysis was performed to identify the optimal threshold of the pNMS ablative rate for predicting seizure freedom. The maximum Youden’s index was achieved at a threshold of 39.79%, yielding a sensitivity of 0.95, specificity of 0.56, and AUC of 0.74 (Fig. 3C). At an imbalance ratio of 0.7, the BAcc was 0.83, which was significantly different from the chance level. Importantly, seizure outcomes differed significantly between subgroups dichotomized by this threshold (Pearson *χ²* = 10.16, *P* = 0.001), whereas the RCS-derived inflection point of 58.65% did not reach statistical significance (Pearson *χ²* = 0.92, *P* = 0.34; Fig. 3D). These findings suggest that while the RCS model provides a biologically meaningful reference point, Youden’s index may offer a more clinically actionable threshold. Representative examples illustrating the spatial overlap between the pNMS and ablation cavity for patients with different outcomes are shown in Fig. 3E, and the full cohort overlays are provided in Supplementary Fig. 2. Overall, an optimal ablative rate threshold of 39.79% for the pNMS on the hippocampus appears predictive of seizure freedom and may serve as a clinically informative marker in MRgLITT planning.

### Analyzing structural variables for predicting and interpreting pNMS

**Fig. 4:**
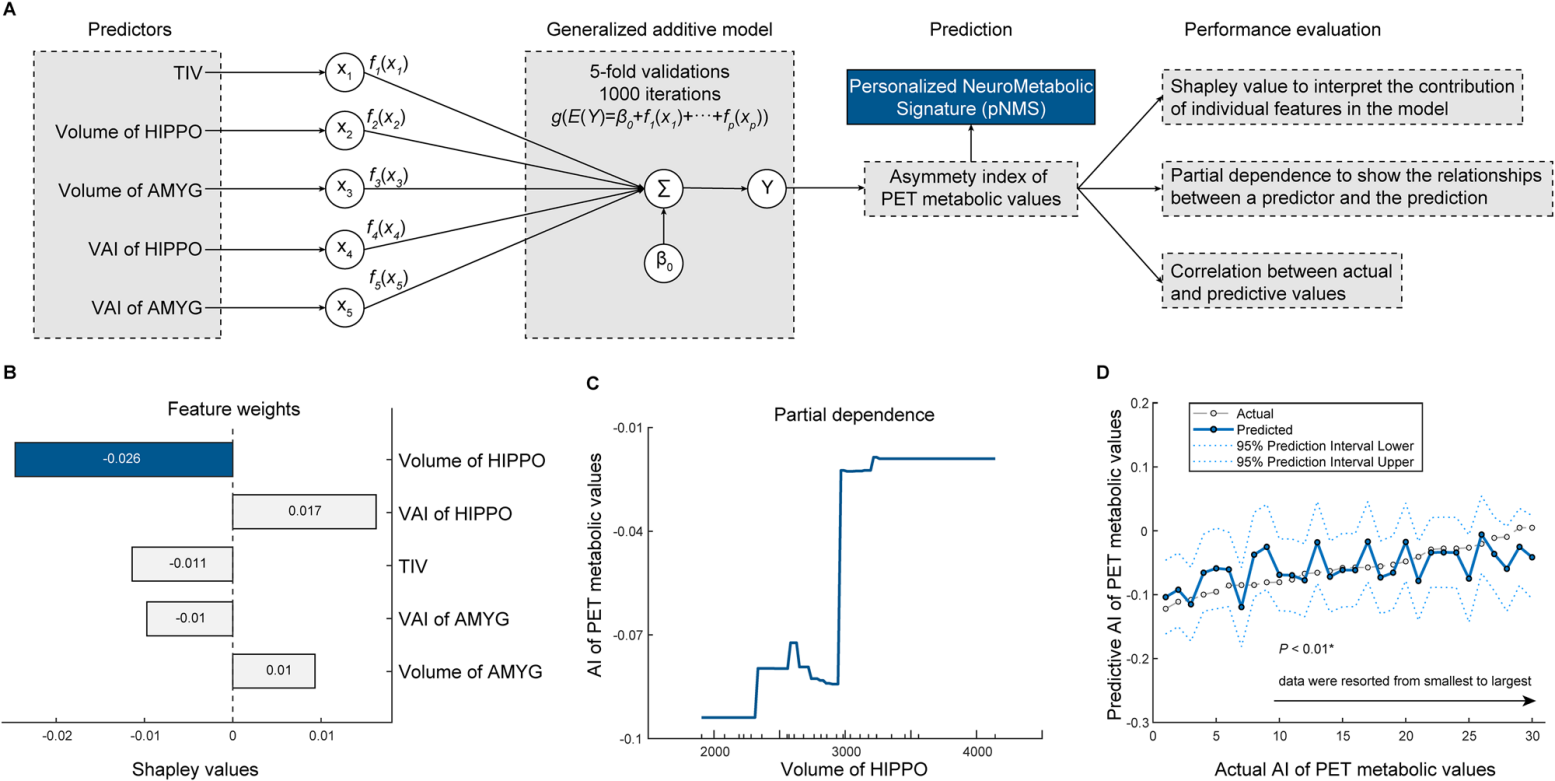
Interpretation of personalized NeuroMetabolic Signature (pNMS) from structural imaging features. **A** Workflow of predictor selection, model construction, and performance evaluation; **B** Shapley values showing the feature weights of each predictor. Larger absolute values indicate greater predictive contribution; **C** Partial dependence plot illustrating the relationship between volume of hippocampus and asymmetry index (AI) of PET metabolic values in the generalized additive model (GAM) model with 1000 bootstrap iterations; **D** Correlation between actual and predictive AI of PET metabolic values. Cases on the X-axis are ranked by predicted values from lowest to highest. The correlation between actual and predicted values with 95% prediction intervals (two-sided Pearson’s correlation, no multiple-comparison correction). All analyses were performed in the full cohort (*n* = 30). * Indicates statistical significance at *P* < 0.05. Abbreviations: HIPPO hippocampus, VAI volumetric asymmetry index, AMYG amygdala, TIV total intracranial volume.

The predictor selection, model construction, and performance evaluation are shown in Fig. 4A. According to Shapley value analysis, the volume of hippocampus had the highest feature weight (−0.026), followed by the VAI of hippocampus, TIV, volume of amygdala, and VAI of amygdala (Fig. 4B). The partial dependence plot shows the marginal relationship between hippocampal volume and the AI of PET metabolic values within the GAM model, indicating that greater asymmetry of the PET signal is generally associated with a smaller ipsilateral hippocampal volume (Fig. 4C). Additionally, a significant correlation was observed between the actual and predicted AI of PET metabolic values (Pearson’s *r* = 0.47, *P* < 0.01; Fig. 4D). These findings suggest that hippocampal atrophy is the principal structural correlate of PET hypometabolic asymmetry and contributes to the development of pNMS, reflecting a structural-functional mapping relationship in mTLE.

## Discussion

In this study, we proposed and explored pNMS as a reliable imaging predictor of seizure outcomes in patients with mTLE who underwent MRgLITT. We demonstrated that the AI of PET metabolic values had the potential to predict seizure outcomes and established its threshold to develop pNMS. We explored an optimal ablative rate threshold of 39.79% for pNMS, which was associated with seizure freedom. Structural analysis also revealed that hippocampal atrophy contributes to the formation of pNMS, suggesting an underlying structure-function relationship.

Our findings provide insights into individualized ablation planning guided by quantitative neuroimaging in patients with mTLE. Currently, surgical planning for MRgLITT typically aims to traverse the medial temporal structures while avoiding the critical vasculature and ventricular system^18^. However, the optimal extent, volume, and specific anatomical targets for ablation remain undefined and are subjects of ongoing debate. Some studies have reported a significant association between total ablation volume and seizure freedom^18–20^ and others have shown that the extent of posterior hippocampal resection does not influence the outcome^21^. Conversely, other studies suggest that sparing the mesial hippocampal head may be associated with persistent seizures^22^. Additional studies have emphasized the role of extra-hippocampal regions, such as the parahippocampal gyrus^23^, piriform cortex^24,25^, and entorhinal cortex^6^, in achieving seizure freedom rather than solely on the amygdalohippocampal complex. A notable study by Jamiolkowski et al. further highlighted the fasciola cinereum, a subregion of the hippocampal tail, as a potential interventional target in epilepsy surgery^26^. These findings collectively suggest that a one-size-fits-all anatomical approach may fail to accommodate the individual variability of the EZ, underscoring the need for personalized, functionally informed strategies such as the proposed pNMS-guided approach.

Previous studies have increasingly explored the use of personalized biomarkers to characterize disease phenotypes. For example, Sara et al. identified hippocampal thickness deviation maps in patients with mTLE by comparing the hippocampal profiles of healthy controls^27^. While this approach enhances the understanding of individual disease characteristics, it relies heavily on external normative datasets, and the variability of control cohorts can introduce uncertainty in interpretation^10^. [^18^F]FDG PET imaging plays a crucial role in localizing the EZ in mTLE due to its ability to represent functional deficits^28,29^. Compared to earlier studies that assessed PET hypometabolism as a prognostic factor using qualitative or semi-quantitative methods^30–32^, our pNMS approach has two key contributions. First, by quantifying inter-hemispheric metabolic asymmetry, our method provides a more specific and personalized diagnostic marker that identifies the EZ of each patient, leading to more accurate and effective ablation strategies. The intra-individual asymmetry approach reduces dependence on group-based comparisons or absolute thresholds, thereby improving generalizability and clinical feasibility^10^. Second, pNMS allows the construction of a spatially individualized mask of metabolic abnormalities, enabling precise quantification of the extent of thermal ablation of the abnormal region, thus bridging metabolic imaging with surgical planning in a patient-specific manner. This individualized ablative target may also be more adaptable to cases with MRI-negative findings, where structural guidance is limited^32^.

Hippocampal atrophy is a well-established feature of mTLE and is closely linked to hypometabolism in the affected regions^33^, with structural atrophy often preceding measurable metabolic decline^34,35^. The loss of neurons and synaptic density leads to reduced glucose metabolism^36^, and chronic neuroinflammation may further exacerbate these deficits^37^. These pathological processes likely contribute to disrupted structural connectivity and impaired functional integration, aligning with our finding that hippocampal atrophy is a key structural correlate of PET-based metabolic asymmetry in patients with mTLE. Overall, the pNMS framework offers a clinically applicable bridge between quantitative functional imaging and personalized surgical targeting, particularly in cases where anatomical landmarks are subtle, absent, or highly variable across individuals.

This study had several limitations. First, the relatively small sample size may limit the generalizability of our findings. Considering the emerging nature of MRgLITT, our cohort of 30 patients with a 1-year postoperative follow-up represents a relatively large sample within this clinical context. These preliminary results provide early evidence to inform future strategies for ablation. Second, the retrospective design introduced the potential for selection bias. Future investigations with larger, prospective cohorts are warranted to validate the utility of pNMS and assess its applicability across broader clinical contexts. In addition, our current analysis focused on the amygdala and hippocampus, consistent with the typical extent of MRgLITT in mTLE patients. However, we acknowledge that hypometabolism in extratemporal regions may also influence postoperative outcomes of epilepsy surgery. Future iterations of the pNMS framework may benefit from incorporating whole-brain asymmetry index mapping, particularly in patients undergoing broader resections involving the piriform cortex, parahippocampal gyrus, and entorhinal cortex.

The proposed pNMS framework offers a clinically relevant method for optimizing ablation strategies in mTLE by integrating PET imaging with individualized targeting. Further validation in multicenter, prospective cohorts is warranted, and mechanistic exploration of pNMS may inform future therapies.

## Supplementary information

Supplement Materials

## Data Availability

Analyzed quantitative code and source data are available within the manuscript and at our GitHub repository (https://github.com/EpiNeuroSurg/pNMS; archived on Zenodo at 10.5281/zenodo.17042331^38^).

## Code availability

All analysis scripts used in this study are available at our GitHub repository (https://github.com/EpiNeuroSurg/pNMS; archived on Zenodo at 10.5281/zenodo.17042331^38^).
