# Supplementary material for: Personalized neurometabolic signature predicts seizure outcomes of laser ablation in mesial temporal lobe epilepsy
Source: Commun Med (Lond). 2025 Nov 20;5:488. doi: 10.1038/s43856-025-01167-0 (PMC12635162; doi:10.1038/s43856-025-01167-0)
Supplement: Supplementary file 1 — Supplement Materials [file 43856_2025_1167_MOESM1_ESM.pdf]

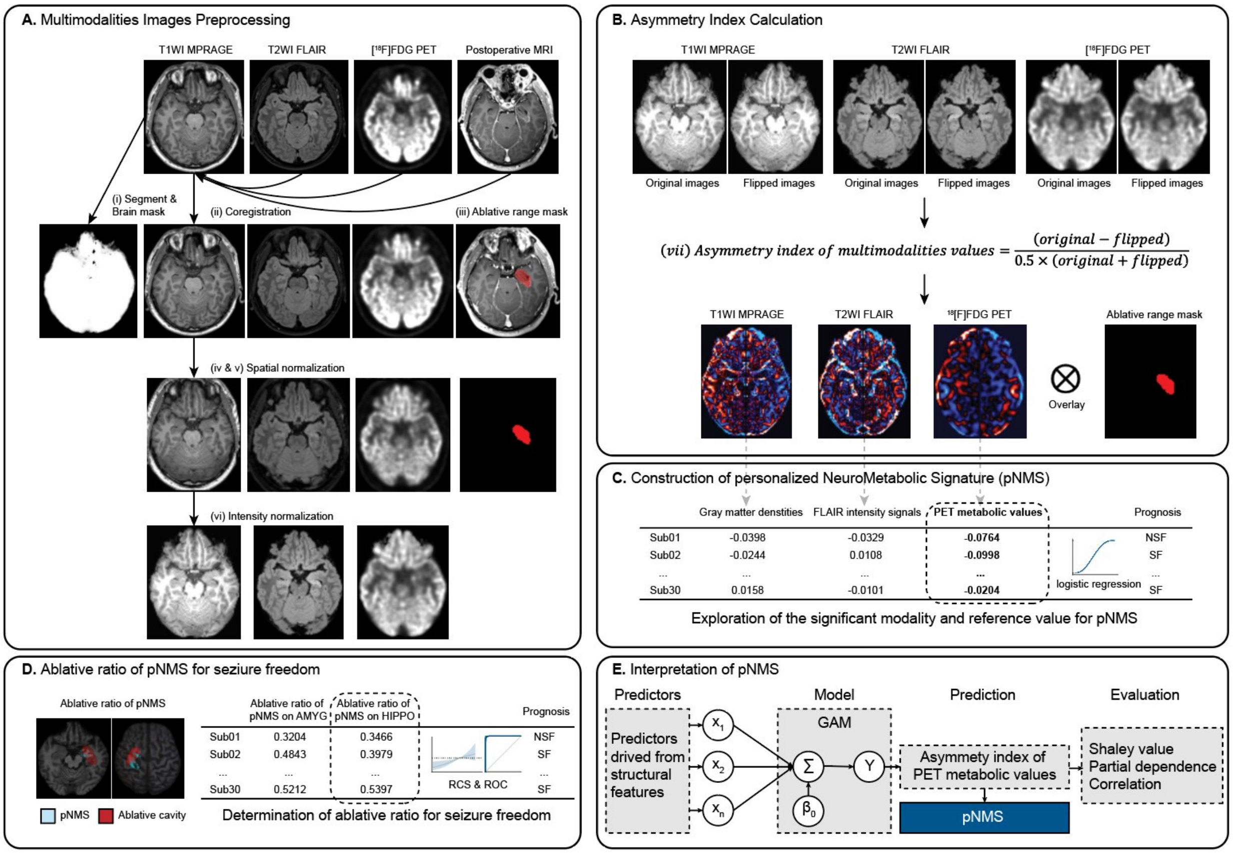

Supplementary Figure 1. Schematic workflow for the construction, validation, and interpretation of the personalized NeuroMetabolic Signature (pNMS). (A) Multimodal neuroimage preprocessing pipeline, including brain segmentation, brain mask generation, coregistration to T1WI images, manual delineation of the ablative cavity, and spatial and intensity normalization of all images; (B) Calculation of asymmetry index (AI) maps of each modality within the ablative range, based on flipped-image subtraction; (C) Quantification of AI values across modalities within the ablative range for each subject. Modalities showing significant group-level differences between seizure-free (SF) and not seizure-free (NSF) groups were considered prognostically informative and used to construct the pNMS using a data-driven thresholding approach; (D) Identification of the optimal ablative ratio of the pNMS associated with seizure freedom; (E) Structural imaging features were input into a generalized additive model (GAM) to predict pNMS expression and investigate structure-function relationships.

Abbreviations: AMYG, amygdala; HIPPO, hippocampus; RCS, restricted cubic spline; ROC, receiver operating characteristic.

Supplementary Table 1. Main and sensitivity analyses of comparing modalities values between seizure freedom (SF) and not seizure freedom (NSF) groups

Variables	Total (n = 30)	NSF (n = 9)	SF (n = 21)	Statistic
**No adjusted covariates**				
T1WI gray matter densities	-0.02 (-0.03, -0.01)	-0.02 (-0.03, -0.01)	-0.02 (-0.03, -0.01)	Mann-Whitney U = -0.00, P = 1.00
T2WI FLAIR signals	0.01 (0.00, 0.02)	0.02 (0.01, 0.03)	0.01 (0.00, 0.01)	Mann-Whitney U = -1.96, P = 0.05
[^{18}F]FDG PET metabolic values	-0.06 ± 0.03	-0.08 ± 0.02	-0.05 ± 0.03	Student's t = -2.59, P = 0.02*
**Adjusted TIV as covariate**				
T1WI gray matter densities	/	/	/	Adjusted R Squared = -0.06, F = 0.15, P = 0.86
T2WI FLAIR signals	/	/	/	Adjusted R Squared = 0.02, F = 1.29, P = 0.29
[^{18}F]FDG PET metabolic values	/	/	/	Adjusted R Squared = 0.19, F = 4.38, P = 0.02*
**Adjusted volume of hippocampus and amygdala as covariate**				
T1WI gray matter densities	/	/	/	Adjusted R Squared = -0.05, F =0.30, P = 0.74
T2WI FLAIR signals	/	/	/	Adjusted R Squared = 0.09, F = 2.36, P = 0.11
[^{18}F]FDG PET metabolic values	/	/	/	Adjusted R Squared = 0.35, F = 8.85, P = 0.001*
**Adjusted asymmetric index of hippocampus and amygdala as covariate**				
T1WI gray matter densities	/	/	/	Adjusted R Squared = -0.03, F = 0.54, P = 0.59
T2WI FLAIR signals	/	/	/	Adjusted R Squared = 0.06, F = 1.89, P = 0.17
[^{18}F]FDG PET metabolic values	/	/	/	Adjusted R Squared = 0.18, F = 4.25, P = 0.03*

Continuous variables with a normal distribution were represented as mean ± standard deviation, and compared using a two-sided independent two-sample t test. For non-normally distributed data, values were reported as median (1st quartile, 3rd quartile), and group comparisons were performed using the two-sided Mann-Whitney test. Between-group differences in [^{18}F]FDG PET metabolic values remained statistically significant in the unadjusted analysis (P = 0.02), when adjusted for TIV (P = 0.02), hippocampal and amygdalar volumes (P = 0.001), or hippocampal and amygdalar asymmetry indices (P = 0.03). * Indicates statistical significance at $P < 0.05$.

Abbreviations: FLAIR, fluid-attenuated inversion recovery; [^{18}F]FDG PET, [^{18}F]fluorodeoxyglucose positron emission tomography; TIV, total intracranial volume.

Supplementary Table 2. Univariate logistic regression analysis of predictors for seizure freedom (*n* = 30)

Variables	*β*	*S.E*	*Z*	*P*	ORs (95% CIs)
Side of EZ					
Right					1.00 (Reference)
Left	-0.41	0.83	-0.49	0.63	0.67 (0.13–3.41)
SEEG implantation					
No					1.00 (Reference)
Yes	-1.22	0.87	-1.40	0.16	0.29 (0.05–1.62)
Age at seizure onset (yrs)	-0.04	0.05	-0.80	0.42	0.96 (0.88–1.06)
Epilepsy duration (yrs)	-0.03	0.04	-0.74	0.47	0.97 (0.89–1.05)
Absolute volume of ablative cavity	0.00	0.00	1.79	0.07	1.00 (1.00–1.00)
Normalized volume of ablative cavity	0.35	0.22	1.60	0.11	1.41 (0.92–2.16)
AI of T1WI gray matter densities	0.08	0.15	0.56	0.58	1.09 (0.81–1.47)
AI of T2WI FLAIR signals	-0.29	0.21	-1.40	0.16	0.75 (0.50–1.12)
AI of [^{18}F]FDG PET metabolic values	0.36	0.16	2.17	0.03*	1.43 (1.04–1.97)

Odds ratios (ORs) with 95% confidence intervals (CIs) were calculated using two-sided logistic regression models. Among all predictors, only the asymmetry index (AI) of [^{18}F]FDG PET metabolic values was significantly associated with seizure freedom (*P* = 0.03). * Indicates statistical significance at *P* < 0.05.

Abbreviations: EZ, epileptogenic zone; SEEG, stereoelectroencephalography; FLAIR, fluid-attenuated inversion recovery; AI, asymmetry index; [^{18}F]FDG PET, [^{18}F]fluorodeoxyglucose positron emission tomography.

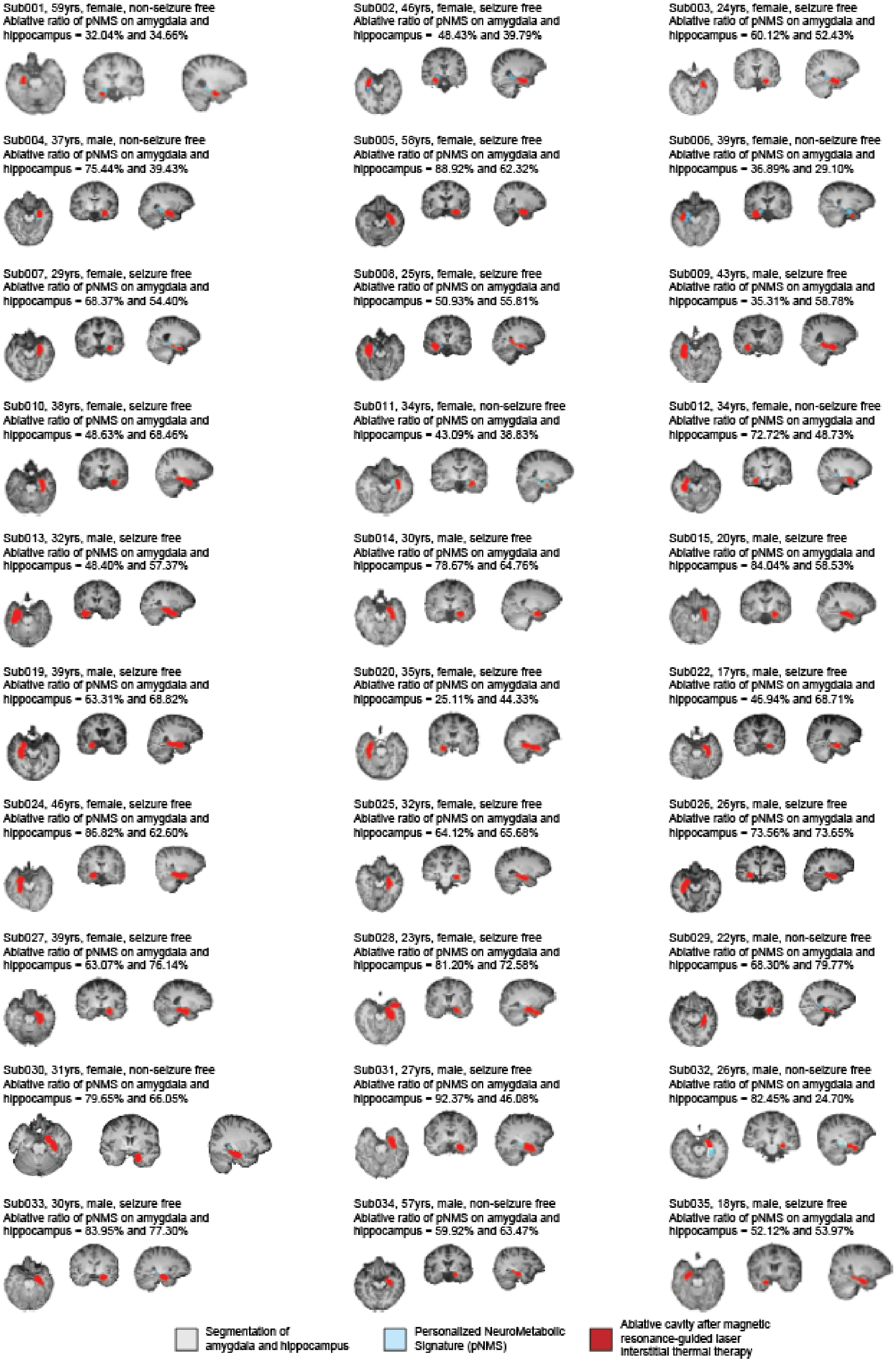
Sub001, 59yrs, female, non-seizure free
Ablative ratio of pNMS on amygdala and hippocampus = 32.04% and 34.66%
Sub002, 46yrs, female, seizure free
Ablative ratio of pNMS on amygdala and hippocampus = 48.43% and 39.79%
Sub003, 24yrs, female, seizure free
Ablative ratio of pNMS on amygdala and hippocampus = 60.12% and 52.43%
Sub004, 37yrs, male, non-seizure free
Ablative ratio of pNMS on amygdala and hippocampus = 75.44% and 39.43%
Sub005, 56yrs, female, seizure free
Ablative ratio of pNMS on amygdala and hippocampus = 88.92% and 62.32%
Sub006, 39yrs, female, non-seizure free
Ablative ratio of pNMS on amygdala and hippocampus = 36.89% and 29.10%
Sub007, 29yrs, female, seizure free
Ablative ratio of pNMS on amygdala and hippocampus = 68.37% and 54.40%
Sub008, 25yrs, female, seizure free
Ablative ratio of pNMS on amygdala and hippocampus = 50.93% and 55.81%
Sub009, 43yrs, male, seizure free
Ablative ratio of pNMS on amygdala and hippocampus = 35.31% and 58.78%
Sub010, 38yrs, female, seizure free
Ablative ratio of pNMS on amygdala and hippocampus = 48.63% and 68.46%
Sub011, 34yrs, female, non-seizure free
Ablative ratio of pNMS on amygdala and hippocampus = 43.09% and 38.83%
Sub012, 34yrs, female, non-seizure free
Ablative ratio of pNMS on amygdala and hippocampus = 72.72% and 48.73%
Sub013, 32yrs, male, seizure free
Ablative ratio of pNMS on amygdala and hippocampus = 48.40% and 57.37%
Sub014, 30yrs, male, seizure free
Ablative ratio of pNMS on amygdala and hippocampus = 78.67% and 64.76%
Sub015, 20yrs, male, seizure free
Ablative ratio of pNMS on amygdala and hippocampus = 84.04% and 58.53%
Sub019, 39yrs, male, seizure free
Ablative ratio of pNMS on amygdala and hippocampus = 63.31% and 68.82%
Sub020, 35yrs, female, seizure free
Ablative ratio of pNMS on amygdala and hippocampus = 25.11% and 44.33%
Sub022, 17yrs, male, seizure free
Ablative ratio of pNMS on amygdala and hippocampus = 46.94% and 68.71%
Sub024, 46yrs, female, seizure free
Ablative ratio of pNMS on amygdala and hippocampus = 86.82% and 62.60%
Sub025, 32yrs, female, seizure free
Ablative ratio of pNMS on amygdala and hippocampus = 64.12% and 65.68%
Sub026, 26yrs, male, seizure free
Ablative ratio of pNMS on amygdala and hippocampus = 73.56% and 73.65%
Sub027, 39yrs, female, seizure free
Ablative ratio of pNMS on amygdala and hippocampus = 63.07% and 76.14%
Sub028, 23yrs, female, seizure free
Ablative ratio of pNMS on amygdala and hippocampus = 81.20% and 72.58%
Sub029, 22yrs, male, non-seizure free
Ablative ratio of pNMS on amygdala and hippocampus = 68.30% and 79.77%
Sub030, 31yrs, female, non-seizure free
Ablative ratio of pNMS on amygdala and hippocampus = 79.65% and 66.05%
Sub031, 27yrs, male, seizure free
Ablative ratio of pNMS on amygdala and hippocampus = 92.37% and 46.08%
Sub032, 26yrs, male, non-seizure free
Ablative ratio of pNMS on amygdala and hippocampus = 82.45% and 24.70%
Sub033, 30yrs, male, seizure free
Ablative ratio of pNMS on amygdala and hippocampus = 83.95% and 77.30%
Sub034, 57yrs, male, non-seizure free
Ablative ratio of pNMS on amygdala and hippocampus = 59.92% and 63.47%
Sub035, 18yrs, male, seizure free
Ablative ratio of pNMS on amygdala and hippocampus = 52.12% and 53.97%
Segmentation of amygdala and hippocampus
Personalized NeuroMetabolic Signature (pNMS)
Ablative cavity after magnetic resonance-guided laser interstitial thermal therapy

Supplementary Figure 2. Overlay relationship between the personalized NeuroMetabolic Signature (pNMS) and the ablative cavity of each patient (*n* = 30). Grey, blue and red ranges indicated the overlay relationship between individual segmentation of amygdala and hippocampus, pNMS, and ablative cavity after magnetic resonance-guided laser interstitial thermal therapy (MRgLITT).

Abbreviations: AMYG, amygdala; HIPPO, hippocampus.
